# Tumor size as a significant prognostic factor in T1 gastric cancer: a Surveillance, Epidemiology, and End Results (SEER) database analysis

**DOI:** 10.1186/s12876-023-02737-z

**Published:** 2023-04-12

**Authors:** Xueyan Xiao, Beibei Gao, Suya Pang, Zeyu Wang, Weiwei Jiang, Weijun Wang, Rong Lin

**Affiliations:** 1 Department of Gastroenterology, Union Hospital, Tongji Medical College, Huazhong University of Science and Technology, Wuhan, 430022 China; 2 Department of Pathology, Union Hospital, Tongji Medical College, Huazhong University of Science and Technology, Wuhan, 430022 China

**Keywords:** SEER program, Stomach Neoplasms, Prognosis, Nomograms; tumor size

## Abstract

**Background:**

It has previously been observed that the prognostic value of tumor size varied according to different stages patients enrolled in gastric cancer. We aimed to investigate the influence of T stage on the prognostic and predicting value of tumor size.

**Material and methods:**

A total of 13,585 patients with stage I–III gastric cancer were selected from the Surveillance, Epidemiology, and End Results Program (SEER) database. Univariate and multivariate cox regression analysis stratified by T stage were performed. C-index and time-dependent receiver operating characteristic curve (ROC) curve were applied to assess discrimination ability of tumor size and other factors. Nomograms were constructed to further assess the performance of tumor size in a specific model. Calibration ability, discrimination ability, reclassification ability and clinical benefits were executed to judge the performance of models.

**Results:**

Stratified analyses according to T stage illustrated that with the increase of T stage, the effect of tumor size on overall survival (OS) and cancer-specific survival (CSS) significantly decreased. Moreover, tumor size showed superior discrimination ability in T1 gastric cancer, outperformed other prognostic factors in predicting both CSS (C-index: 0.666, AUC: 0.687) and OS (C-index: 0.635, AUC: 0.660). The cox regression model included tumor size showed better performance than the model excluded tumor size in every aspect.

**Conclusion:**

T stage had a negative impact on the predicting value of tumor size. Tumor size showed significant prognostic value in T1 gastric cancer, which may be effective in clinical practice.

**Supplementary Information:**

The online version contains supplementary material available at 10.1186/s12876-023-02737-z.

## Introduction

Gastric cancer, as the fifth frequent cancer and the third primary cause of cancer death in the world, has been greatly concerned [1]. Especially in China, though the 5-year survival improved enormously in the past 20 years, it was still about 35% from 2010–2014 [2]. Nowadays, the American Joint Committee on Cancer (AJCC) tumor-node-metastasis (TNM) staging system is widely used for the diagnosis and risk stratification of gastric cancer [3]. The tumor infiltration depth (T stage), lymph node metastasis (N stage), and tumor metastasis (M stage) were the mainly prognostic factors which has been widely acknowledged. Whereas, even patients in the same TNM stage showed different prognosis. Lin et al. analyzed the prognostic value of the eight edition AJCC TNM staging classification for gastric cancer, and discovered that the subgroups in stage III illustrated significantly different 5-year OS rates [4]. Bando et al. found that the overall survival displayed heterogeneous distribution in the same AJCC stage, which may cause undertreatment or unnecessary overtreatment [5]. Therefore, to further classify the stages, additional valued prognostic factors were being sought.

Tumor size, which is specified as the maximal horizontal diameter of tumor, may be an alternative option not only because of its measurability and accessibility before or during surgery [6], but also owing to its significant relevance to survival. It has already applied in the in the AJCC T staging for several cancer such as lung cancer, liver cancer and breast cancer [3]. There were also quite a lot of researches focused on the prognostic value of tumor size in gastric cancer [7–9]. While the prognostic value of tumor size was still unclear. Several articles confirmed that tumor size was a non-negligible prognostic factor in gastric cancer and could improve the accuracy of survival prediction [7, 10, 11]. However, the multi-variable regression model conducted in a few articles indicated tumor size was not significant in gastric cancer to predict survival [12–15]. What is instructive to explain the above problem is that Im et al. investigated that tumor size only showed significant prognostic predicting value in advanced gastric cancer [16]. Liu et al. also proposed that the evaluation of prognostic value of tumor size may be practicable only when depth of infiltration was specified [17].

Thus, we aimed to investigate whether T stage had an impact on the predicting ability of tumor size in gastric cancer and intended to explore whether tumor size has a convincing prognostic predicting ability and can truly improve the accuracy of prognostic predictions in the Surveillance, Epidemiology, and End Results (SEER) database.

## Methods

We obtained data from The Surveillance, Epidemiology, and End Results (SEER) Program (https://seer.cancer.gov/), and identified 40,836 patients aged 18 years and older in total, who were pathologically diagnosed with gastric cancer between 2004 and 2018. The explicit criteria for patient selection and data processing can be seen in (See Additional file 1). All data generated or analyzed during this study are included in this published article and its supplementary information files named “analysis data”.

Variables significantly associated with CSS and OS were identified by univariate and multivariate cox regression models. Tumor size was analyzed as continuous variable, and the other were analyzed as categorical variables. The concordance index (C-index) and ROC curve [18] were utilized to assess the discrimination ability of tumor size and other factors.

Then we constructed nomograms based on multivariate cox regression which illustrated the relationship between specific clinical prognostic factors and CSS or OS. We chose the quartiles of tumor size in T1 stage to be the cut-off value, which classified tumor size into four parts: ≤ 1.2 cm; 1.2–2.1 cm; 2.1–3.7 cm; ≥ 3.7 cm. The performance between the models with tumor size included and not included was evaluated. The details can be seen in Additional file 1.

All the above statistical analyses were performed with SPSS, version 22.0 (SPSS, Chicago, IL, RRID:SCR_002865) and R (R version 4.0.4, www.r-project.org, RRID:SCR_001905). We considered two-sided *p*-values less than 0.05 to be statistically significant.

## Results

### Patient characteristics

There were 13,585 patients from the SEER database enrolled in our study. A total of 6848(50.4%) patients were less than 68 years old. Of the patients, 8815(64.9%) were female, 9491(69.9%) were white and 8178(60.2%) were married. More detailed demographic and clinical characteristics information was listed in Table 1.

**Table 1:** Baseline clinicopathological features stratified by T stage

Factor	All	T1	T2	T3	T4
**Age**					
< 68	6848(50.4%)	1586(44.7%)	965(48.4%)	2968(54.0%)	1329(52.2%)
≥ 68	6737(49.6%)	1965(55.3%)	1030(51.6%)	2526(46.0%)	1216(47.8%)
**Gender**					
Male	8815(64.9%)	2210(62.2%)	1315(65.9%)	3790(69.0%)	1500(58.9%)
Female	4770(35.1%)	1341(37.8%)	680(34.1%)	1704(31.0%)	1045(41.1%)
**Ethnicity**					
White	9491(69.9%)	2416(68.0%)	1419(71.1%)	4032(73.4%)	1624(63.8%)
Black	1638(12.1%)	412(11.6%)	236(11.8%)	611(11.1%)	379(14.9%)
Others	2456(18.1%)	723(20.4%)	340(17.0%)	851(15.5%)	542(21.3%)
**Marital Status**					
Single	1850(13.6%)	455(12.8%)	230(11.5%)	771(14.0%)	394(15.5%)
Married	8178(60.2%)	2073(58.4%)	1243(62.3%)	3399(61.9%)	1463(57.5%)
Widowed	1796(13.2%)	540(15.2%)	277(13.9%)	629(11.4%)	350(13.8%)
Divorced	1267(9.3%)	339(9.5%)	172(8.6%)	508(9.2%)	248(9.7%)
Unknown	494(3.6%)	144(4.1%)	73(3.7%)	187(3.4%)	90(3.5%)
**Site of cancer**					
proximal	5114(37.6%)	1199(33.8%)	772(38.7%)	2613(47.6%)	530(20.8%)
middle	3546(26.1%)	1037(29.2%)	539(27.0%)	1217(22.2%)	753(29.6%)
distal	3392(25.0%)	958(27.0%)	497(24.9%)	1127(20.5%)	810(31.8%)
overlapping	729(5.4%)	137(3.9%)	86(4.3%)	274(5.0%)	232(9.1%)
Stomach, NOS	804(5.9%)	220(6.2%)	101(5.1%)	263(4.8%)	220(8.6%)
**Grade**					
I	684(5.0%)	371(10.4%)	96(4.8%)	161(2.9%)	56(2.2%)
II	3710(27.3%)	1152(32.4%)	639(32.0%)	1450(26.4%)	469(18.4%)
III/IV	8216(60.5%)	1700(47.9%)	1127(56.5%)	3506(63.8%)	1883(74.0%)
Unknown	975(7.2%)	328(9.2%)	133(6.7%)	377(6.9%)	137(5.4%)
**Histology**					
Adenocarcinoma	9913(73.0%)	2693(75.8%)	1525(76.4%)	4099(74.6%)	1596(62.7%)
Mucinous adenocarcinoma	431(3.2%)	50(1.4%)	68(3.4%)	216(3.9%)	97(3.8%)
Signet ring cell carcinoma	3241(23.9%)	808(22.8%)	402(20.2%)	1179(21.5%)	852(33.5%)
**N stage**					
N0	6402(47.1%)	2911(82.0%)	1104(55.3%)	1603(29.2%)	784(30.8%)
N1	3379(24.9%)	454(12.8%)	563(28.2%)	1796(32.7%)	566(22.2%)
N2	2153(15.8%)	141(4.0%)	220(11.0%)	1240(22.6%)	552(21.7%)
N3	1651(12.2%)	45(1.3%)	108(5.4%)	855(15.6%)	643(25.3%)
**Tumor size**	13,585	3551	1995	5494	2545
**Surgery**					
no surgery	2368(17.4%)	802(22.6%)	332(16.6%)	890(16.2%)	344(13.5%)
Partial or subtotal or hemi- gastrectomy	5505(40.5%)	1576(44.4%)	856(42.9%)	1918(34.9%)	1155(45.4%)
Near-total or total gastrectomy	1432(10.5%)	299(8.4%)	181(9.1%)	625(11.4%)	327(12.8%)
With removal of a portion of esophagus	3153(23.2%)	676(19.0%)	474(23.8%)	1584(28.8%)	419(16.5%)
With the resection of other organs	1047(7.7%)	175(4.9%)	141(7.1%)	439(8.0%)	292(11.5%)
Surgery, NOS	80(0.6%)	23(0.6%)	11(0.6%)	38(0.7%)	8(0.3%)
**LNH**					
None	2691(19.8%)	966(27.2%)	380(19.0%)	945(17.2%)	400(15.7%)
1–3	713(5.2%)	237(6.7%)	104(5.2%)	228(4.1%)	144(5.7%)
≥ 4	9948(73.2%)	2287(64.4%)	1478(74.1%)	4211(76.6%)	1972(77.5%)
lymph nodes removed, NOS	119(0.9%)	32(0.9%)	19(1.0%)	50(0.9%)	18(0.7%)
Unknown	114(0.8%)	29(0.8%)	14(0.7%)	60(1.1%)	11(0.4%)

*LNH* Lymph Node Harvest

### The prognostic value of tumor size varied with T stage

**Fig. 1:**
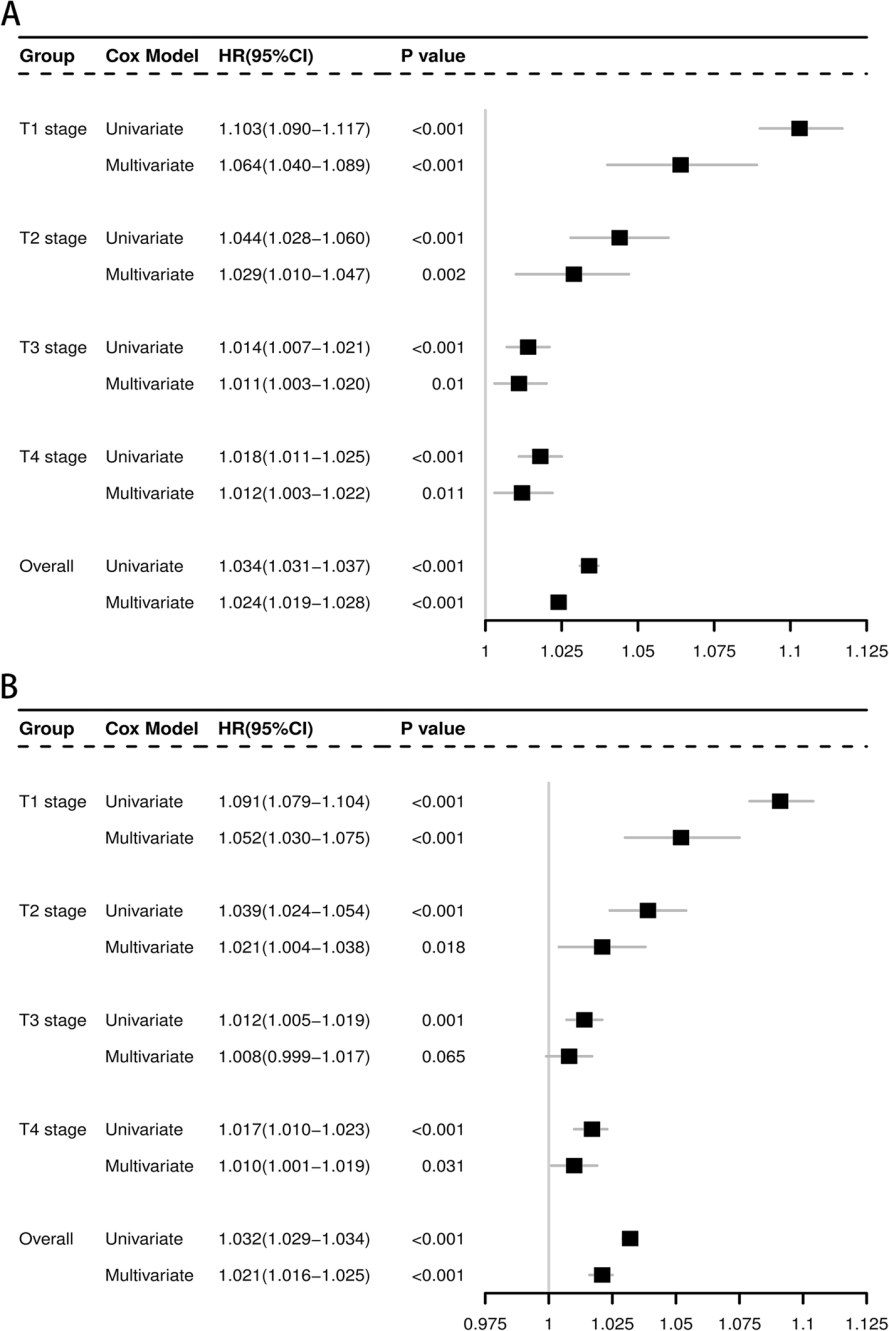
Impact of T stage on the hazard ratio of tumor size in predicting CSS and OS in gastric cancer. **A** CSS in SEER database. **B** OS in SEER database. Abbreviations: CSS, cancer-specific survival; OS, overall survival; SEER, Surveillance, Epidemiology, and End Results

The prognostic value of tumor size was evaluated by univariate and multivariate Cox proportional hazards regression analyses (Additional file 2 and 3). The results conducted in all patients and stratified by T stage were showed in Fig. 1.

As shown in the table, tumor size was an independent prognostic factor both in CSS set and OS set, which was not affected by T stage. The larger the tumor, the higher the risk of death.

It should be noted that hazard ratio (HR), which reflected the death risk, declined significantly with the increase of T stage. The impact of tumor size on risk of death was the strongest in T1 stage (HR: 1.064 in CSS set; 1.052 in OS set). While the prognostic significance of tumor size in T3 (HR: 1.011 in CSS set; 1.008 in OS set) and T4 (HR: 1.012 in CSS set; 1.010 in OS set) stage is similar but both weaker than those in T1 and T2 stages.

### Discriminatory ability of tumor size outperformed in T1 stage

To further evaluate the discrimination ability of tumor size and other factors, we calculated Harrell’s C index and AUC of time-dependent ROC curves. We also plotted a heat map to give visual presentation (Additional file 4). Of all the patients, tumor size was the most important predictive factor for OS (C-index 0.587, AUC 0.635) with the highest C-index and AUC other than surgery and LNH (Additional file 5). However, in CSS set, N stage (C-index 0.587, AUC 0.653) and tumor size (C-index 0.603, AUC 0.649) made no distinction of rank with each other (Table 2). In further analyses stratified by T stage, it is remarkable to find that tumor size showed extremely large discrimination ability for CSS (C-index 0.666, AUC 0.687) and OS (C-index 0.635, AUC 0.660) in and only in T1 stage, which is outstanding than any other factors except for surgery and LNH.

**Table 2:** Discriminatory ability of clinicopathological factors in predicting CSS in gastric cancer

	**Total**		**T1**		**T2**		**T3**		**T4**	
	C-index	AUC	C-index	AUC	C-index	AUC	C-index	AUC	C-index	AUC
**Tumor size**	0.603	0.649	0.666	0.687	0.544	0.555	0.525	0.531	0.541	0.571
**Age**	0.556	0.545	0.597	0.614	0.567	0.571	0.549	0.532	0.572	0.557
**Sex**	0.503	0.518	0.515	0.534	0.510	0.523	0.505	0.519	0.512	0.511
**Race**	0.529	0.539	0.555	0.564	0.542	0.556	0.521	0.537	0.528	0.539
**Marital status**	0.540	0.536	0.581	0.587	0.533	0.528	0.528	0.523	0.544	0.550
**Site**	0.540	0.567	0.575	0.593	0.576	0.615	0.538	0.580	0.528	0.523
**Grade**	0.540	0.551	0.532	0.527	0.506	0.508	0.525	0.519	0.523	0.551
**Histology**	0.500	0.504	0.524	0.528	0.541	0.548	0.504	0.503	0.499	0.554
**N stage**	0.587	0.653	0.537	0.522	0.560	0.596	0.566	0.615	0.516	0.586
**Surgery**	0.612	0.641	0.744	0.792	0.636	0.663	0.583	0.607	0.567	0.591
**LNH**	0.596	0.606	0.713	0.756	0.621	0.634	0.581	0.593	0.576	0.589

*LNH* Lymph Node Harvest, *AUC* Area under the receiver operating characteristic curve (ROC)

In order to exclude the influence of surgery and LNH, patients in T1 stage were separated into two groups based on surgery status and LNH status respectively (Additional file 6). It is found that among all the factors, tumor size achieved best and kept the discrimination ability regardless of surgery or LNH status.

In conclusion, compared to higher T stage, tumor size illustrated much better discrimination ability than any other prognostic factors in T1 stage.

### Construction and comparison of nomogram based on tumor size in T1 gastric cancer

Subsequently, we constructed nomogram to facilitate the application of predicting ability of tumor size in clinic. We also intended to investigate the difference between nomograms included and not included tumor size.

**Fig. 2:**
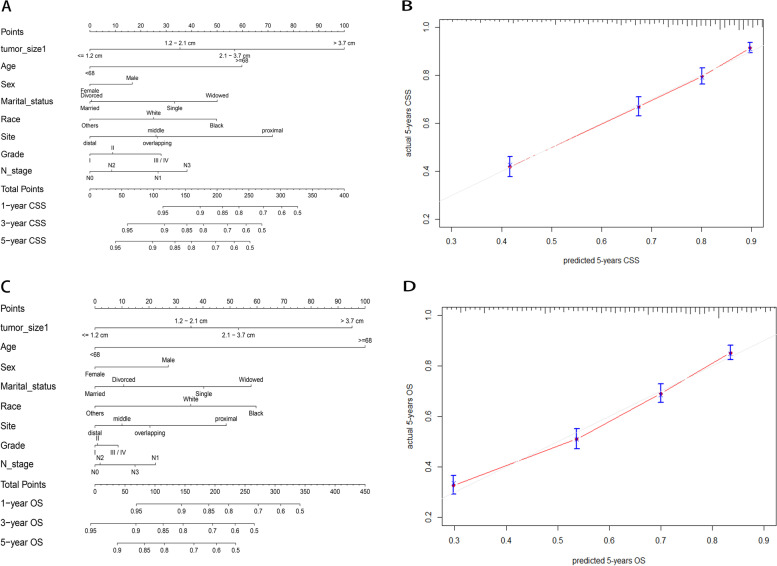
Construction and calibration of tumor size–based nomogram in T1 gastric cancer. **A** Tumor size–based nomogram in CSS set. The nomogram can be used to obtain the probability of one-, three- and five-year survival by adding up the points identified on the point scale for each variable. **B** Calibration curve for tumor size based nomogram in CSS set. The grey line represents the ideal fit; The red line represents the current nomogram; The vertical bars represent the 95% CIs of the estimates. **C** Tumor size–based nomogram in OS set. **D** Calibration curve for tumor size based nomogram in OS set. Abbreviations: SRCC, sigle ring cell carcinoma; MC, Mucinous adenocarcinoma

**Fig. 3:**
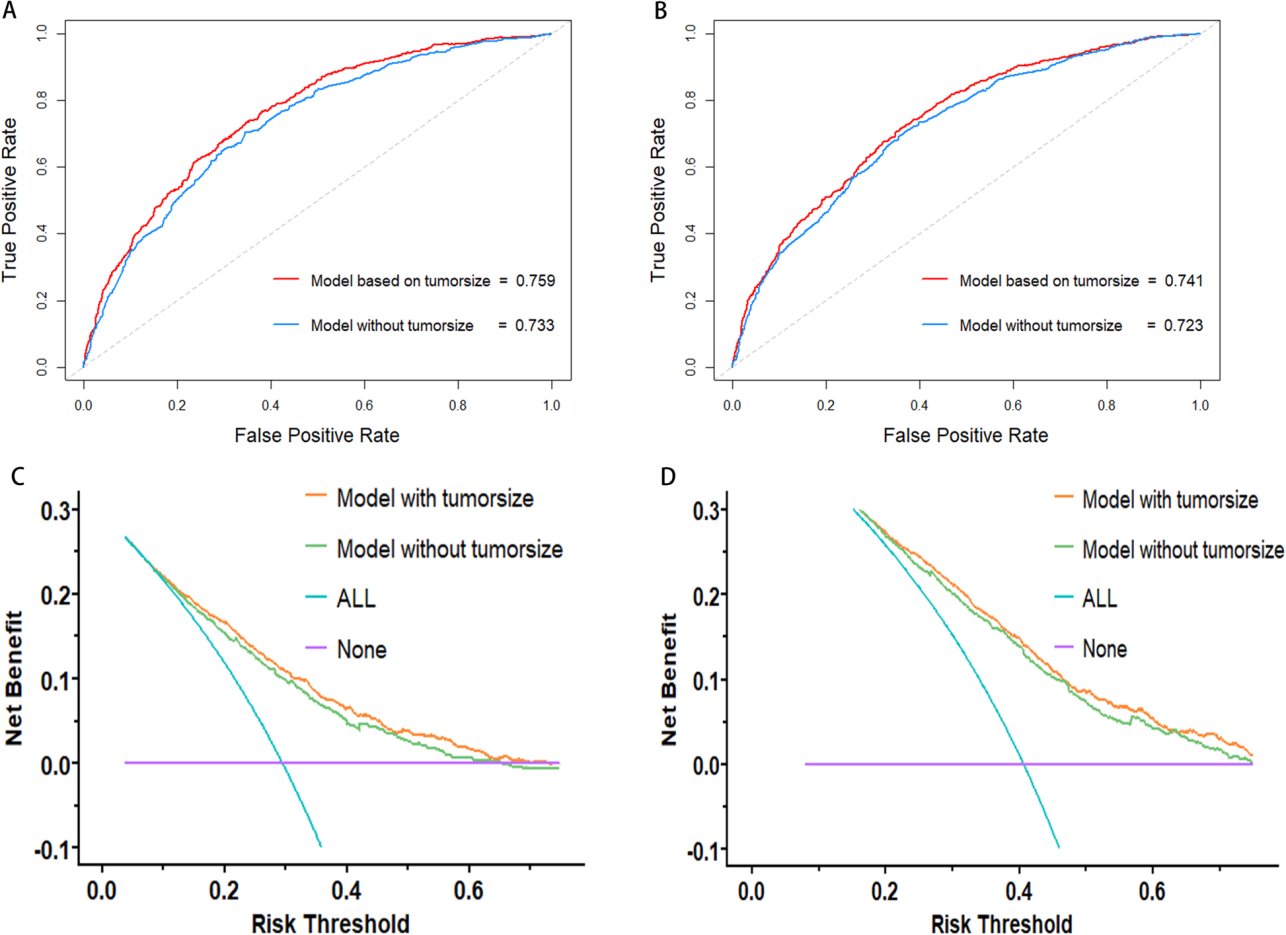
Performance between nomogram with tumor size and nomogram without tumor size in T1 gastric cancer. **A** Time-dependent receiver operating characteristic curve (ROC) of the nomogram with or without tumor size in CSS set. **B** Time-dependent receiver operating characteristic curve (ROC) of the nomogram with or without tumor size in OS set. **C** Decision curve analysis for cancer-specific survival (CSS). **D** Decision curve analysis for overall survival (OS)

The nomograms and calibration curves were showed in Fig. 2. Harrell’s C index of nomograms for CSS and OS prediction was 0. 0.720 (95% CI, 0.702–0.738) and 0.700 (95% CI, 0.685–0.715), respectively (Table 3). Time-dependent ROC curve showed that model with tumor size incorporated indicated better performance in both CSS (AUC at 5 years: 0.759 vs. 0.733) and OS (AUC at 5 years: 0.741 vs 0.723) sets. The ROC curves were plotted in Fig. 3A, B. To further evaluate an added prognostic discrimination power for tumor size, we assessed IDI and NRI (Table 3). A significant improvement can be observed that the proportion of correct classifications increased 17.2% and 15.4% in CSS and OS set, if tumor size was included in the model. The IDI also suggested that model with tumor size produced more accurate predictions (CSS set: 0.026(0.011–0.037), OS set: 0.025(0.010–0.036)). We next exhibited DCA curve to evaluate the clinical usability of the nomogram (Fig. 3C, D). We can see that with tumor size incorporated, the model showed larger net clinical benefits either in the prediction of CSS or OS. The area under DCA curve (AUDC) was calculated to demonstrate the benefit more directly. As shown in Table 3, AUDC increased 0.006 in CSS sets and 0.009 in OS sets.

**Table 3:** Prediction Performance between models with or without tumor size

	**CSS sets**		**OS sets**	
	**Model without tumor size**	**Model with tumor size**	**Model without tumor size**	**Model with tumor size**
**C-index**	0.693(0.674–0.711)	0.720(0.702–0.738)	0.681(0.666–0.696)	0.700(0.685–0.715)
**AUC(5-years)**	0.733	0.759	0.723	0.741
**AUDC**	0.060	0.066	0.095	0.104
**The reclassification ability improved**				
** IDI**	Reference	0.026(0.011–0.037)	Reference	0.025(0.010–0.036)
** NRI**		0.172(0.089–0.299)		0.154(0.095–0.264)
** NRI (events)**		0.159(0.096–0.191)		0.122(0.606–0.155)
** NRI (no events)**		0.014(-0.024–0.166)		0.032(-0.025–0.174)

*CSS* Cancer-specific survival, *OS* Overall survival, *LNH* Lymph Node Harvest, *AUC* Area under the receiver operating characteristic curve (ROC), *AUDC* Area under decision curve analyses (DCA) curve, *IDI* the integrated discrimination improvement, *NRI* the net reclassification improvement

To further intuitively exhibit the prognostic value of tumor size, we plotted survival curves stratified by tumor size in T1-T4 stage respectively (Additional file 7). Tumor size was stratified into four groups based on the quartiles in T1 stage. It can be seen is that only in T1 stage, patients stratified by tumor size showed significant discriminative survival curves. While in T2-T4 stage, the survival curves were overlapped.

## Discussion

Our current study investigated how T stage impact on the prognostic value of tumor size and verified the prognostic predicting value of tumor size in T1 stage. First of all, tumor size was an independent prognostic factor both in CSS set and OS set, which was not affected by T stage. Second, T stage had a negative effect on the prognostic value of tumor size. Third, by comparing the discrimination ability of common clinical factors, tumor size also outperformed, even better than N stage in T1 stage. Fourth, the nomogram with tumor size included exhibited superior discriminative ability, reclassification ability and improved clinical benefits than model without tumor size as well.

There were abundant researches focused on the prognostic factors of gastric cancer, the hottest among which included lymph node status, depth of invasion, curative resection and so on [19–22]. As for the influence of tumor size, it had caused great concern for the reason that tumor size can be easily measured before or during the surgery, especially when endoscopic techniques come into use. Some illustrated the independent prognostic value of tumor size cannot be neglected in gastric cancer [7, 11, 23]. While others found tumor size became insignificant in multivariate analysis of prognostic prediction in gastric cancer [12–15]. A possible reason may be that T stage can influence the prognostic value of tumor size. Some researchers had raised a presumption that the impact of tumor size was practical only when the depth of invasion was specified [9, 17]. However, they didn’t reveal how invasion depth affect the predicting value of tumor size. In our study, we systematically analyzed the changes in HR of tumor size caused by the stratification of T stage and confirmed that T stage did affect the predicting value of tumor size. As the increment of T stage, HR of tumor size decreased. One possible reason could be that the influence of tumor size may be covered in higher stage. As is well-known, the invasion depth had significantly correlation to lymph node metastasis and tumor metastasis, while the horizontal proliferation seemed to be less correlated [24–27]. Once metastasis occurred, the survival of patients sharply declined [21, 28]. Thus, the stronger prognostic value of metastasis presented more commonly in higher stage made the prognostic value of tumor size less important. Another explanation may be the measuring error of tumor size in higher stage, when tumor infiltrated the serosa, it’s hard to define the horizontal diameter, and merely the maximum horizontal diameter cannot represent the tumor grow extent [6].

Furthermore, there were a few researchers studied the predicting value of tumor size was influenced by T stage in colon cancer, esophageal cancer and gastric cancer [6, 29–31]. Dai et al. found in colon cancer that when T stage increased, the influence of tumor size on death and recurrence risk decreases gradually [6]. Zhang et al. found in esophageal cancer that tumor size had the strongest effect on prognosis for T1 classification and the weakest effect for T4 classification [30]. Chen et al. declared that tumor size showed superior the discriminatory ability at T1 stage [31]. While Chen et al. only take a few factors into analyses and didn’t mention the clinical significance based on DCA curves. Due to the fact that gastric cancer along with colon and esophageal cancer are all gastrointestinal cancer, the conclusion derived can corroborate each other and made it more reliable.

Subsequently, we found that C-index of tumor size was outstanding than some widely accepted clinical prognostic factors, even better than N stage in T1 stage. It is well known that lymph node metastasis was an inescapable prognostic factor that indicated the mode and range of surgery [32, 33]. The survival markedly decreased with the increase of the lymph nodes metastasis [34–36]. However, Sekiguchi M et al. found that the prevalence of LNM was rarely low, about 12.3%, in early gastric cancer [37]. Besides, in our study, patients with no lymph nodes metastasis accounted for 82% in T1 stage. Given all about these, the less predicting value of N stage compared to tumor size in T1 stage made sense. A shortage of data in N2 and N3 patients in T1 stage may also be the reason why N stage acted abnormally in the nomogram. Therefore, we still need further research with more patients participate in to compare the predicting value between tumor size and N stage in T1 gastric cancer.

Another important point of our study is that we proved that tumor size improved the accuracy of prognostic predicting nomogram in T1 stage. We introduced NRI and IDI to better assess the reclassification ability of the model when new factors were added, which could compensate the limitation of AUC [38]. Reclassification can be explained as the movement of patients from one risk category to another based on changes of assignment conducted by predicting models [39]. The difference between NRI and IDI is that NRI mainly assessed the reclassification ability at a specific cut point, while IDI considered the overall improvement of the new model. In our study, for patients had expected event occurred (death in our study), NRI increased 15.9% and 12.2% in CSS and OS set respectively, which means the predicted risk increased in the new model, IDI (CSS sets: 2.6%, OS sets: 2.5%) indicated significant improvement of reclassification ability in model with tumor size included, either.

The application of DCA curve is aimed to assess the clinical usefulness of prediction nomogram by calculating the net benefit among different threshold probabilities [40]. For example, when the clinical intervention threshold for a patient with T1 gastric cancer is between 10 to 65% risk for CSS at 5 years, the nomogram with tumor size included gains more net benefit all the time. In a word, the clinical decision based on nomogram with tumor size included would reduce unnecessary treatment as well as undertreatment.

Nonetheless, there are several limitations in our study. First, because of the finite information in the SEER database, there are many prognostic factors we didn’t take in, such as lymph-vascular invasion, specific differentiation type, ulcer condition and so on. Second, which is also the disadvantage of all retrospective studies, we can’t avoid the selection bias completely. Third, the death of T1 gastric cancer is decreasing these years, take the disease-free survival (DFS) as outcome may be more practical. Consequently, the prognostic value of tumor size in T1 gastric cancer may be further assessed under situation of DFS set as outcome. Fourth, all speculations are based on public data, which still need further validation in clinical work.

When considered about the clinical application of tumor size in gastric cancer, it has been brought into the Japanese gastric cancer treatment guidelines 2018 (5th edition) and Chinese Society of Clinical Oncology (CSCO) guideline [32, 41]. It is mainly mentioned in the endoscopic resection and D1 and D1 + lymphadenectomy that tumor size is an important factor to decide the indication of surgery. What is interesting is that the approaches mentioned above are mostly applied in early-stage gastric cancer, cT1aN0M0 and cT1bN0M0, for instance. However, in advanced-stage gastric cancer, the guiding significance of tumor size is negligible [32], which confirmed the effect of T stage on prognostic value of tumor size exactly. Nevertheless, more researches are needed to assess the clinical value of tumor size especially in T1 stage. For example, the extent of gastric resection is determined on the basis of infiltration depth, location or Borrmann classification [32, 41]. The margin of surgery may be reconsidered according to the influence of tumor size. Moreover, further studied are recommended to investigate the guidance significance of tumor size in staging, diagnosis, treatment, follow up and so on.

## Conclusion

T stage had a negative influence on the prognostic and predictive value of tumor size. With the increase of T stage, the hazard ratio of tumor size decreased significantly. The discrimination ability of tumor size is superior to any other clinical factors, even N stage in T1 gastric cancer. More interestingly, tumor size can improve the accuracy of nomogram predicting prognosis of T1 gastric cancer, and may be worthful to guide the staging system in the future.

## Supplementary Information

**Additional file 1.** Material and Methods.

**Additional file 2:** **Supplementary table 1.** Univariate and Multivariate analysis of prognostic factors affecting OS.

**Additional file 3:** **Supplementary table 2.** Univariate and Multivariate analysis of prognostic factors affecting CS.

**Additional file 4:** **Supplementary figure 2.** Heatmap of C-index of clinicopathological factors in predicting CSS and OS in gastric cancer.

**Additional file 5:** **Supplementary table 3.** Discriminatory ability of clinicopathological factors in predicting OS in gastric cancer.

**Additional file 6:** **Supplementary table 4.** C-index of clinicopathological factors in subgroup.

**Additional file 7:** **Supplementary figure 3.** Survival analysis of CSS and OS stratified by tumor size.

**Additional file 8.**

## Data Availability

All data generated or analyzed during this study are included in this published article and its supplementary information files named “analysis data”.
