# Supplementary material for: Tumor size as a significant prognostic factor in T1 gastric cancer: a Surveillance, Epidemiology, and End Results (SEER) database analysis
Source: BMC Gastroenterol. 2023 Apr 12;23:121. doi: 10.1186/s12876-023-02737-z (PMC10091636; doi:10.1186/s12876-023-02737-z)
Supplement: Supplementary file 1 — Additional file 1. Material and Methods. [file 12876_2023_2737_MOESM1_ESM.docx]

**Material and Methods:**

**Patients and Database**

Patients who met the following criteria were included :1) with only one primary tumor, 2) histologically confirmed primary GC (International Classification of Diseases for Oncology, 3rd edition (ICD-O-3) site codes: C16.0-16.9), 3) staged Ⅰ-Ⅲ, 4) with histological type limited to adenocarcinoma (8140, 8210, 8211, 8260, 8261, 8262, 8263), mucinous adenocarcinoma (8480, 8481), and signet-ring cell carcinoma (8490). 5) without any treatment or treated with gastrectomy. We excluded patients as follow: 1) without diagnostic or staging information, 2) without information of tumor size, 3) unfollowed or survival time equal to 0. Finally, 13585 patients were included **(Supplementary figure 1)**.

**Supplementary figure 1 The flow chart of the study population enrollment**


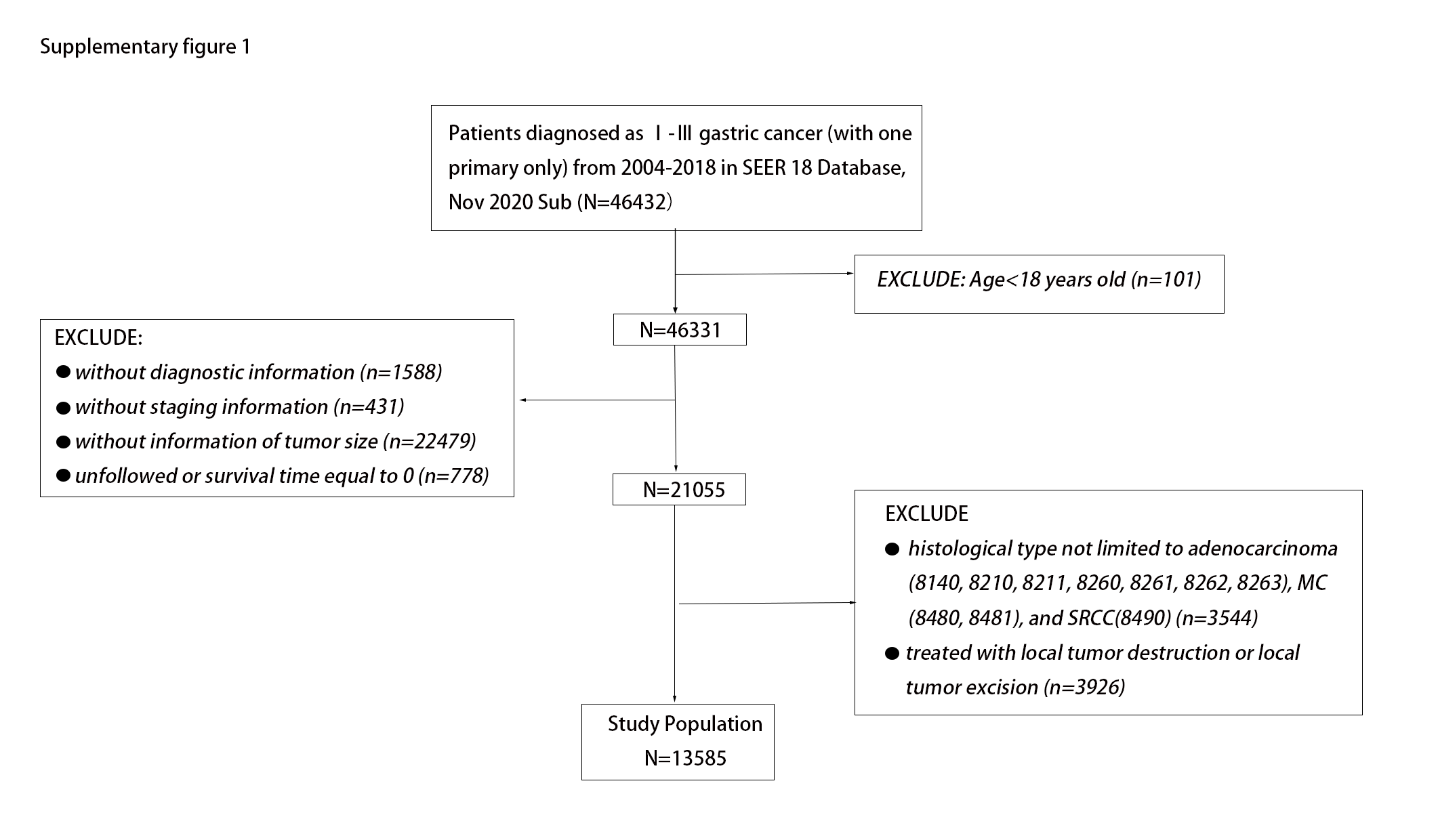


**Data Processing**

The following demographic and clinical characteristics were retrieved from the SEER database based on SEER*Stat software (Version 8.3.9): age, sex, race, marital status, primary site, histology, grade, AJCC stage, T stage, N stage, tumor size, surgery, regional lymph node harvest (LNH), cause of death, survival months. All included patients were restaged based on the AJCC Cancer Staging Manual (8th edition). The cut-off value of age was determined to be at 68 years old, for the reason that age fit the normal distribution and 68 is exactly the average value. The primary site of tumor in the stomach was described as proximal(cardia), middle (fundus, body, lesser curvature, and greater curvature), distal (gastric antrum, pylorus), overlapping (along two locations). Grade is grouped into grade Ⅰ (Well differentiated), grade Ⅱ (Moderately differentiated) and grade Ⅲ/Ⅳ (Poorly differentiated and undifferentiated). Surgery is divided into six groups, no surgery, partial or subtotal or hemi gastrectomy, near-total or total gastrectomy, with removal of a portion of esophagus, with the resection of other organs and surgery, NOS. Lymph node harvest is grouped into none, 1-3 LNH, ≥4 LNH and lymph nodes removed, NOS. Tumor size was analyzed as continuous variable in this study. Our interest of outcomes included cancer specific survival (CSS) and overall survival (OS). CSS was defined as death caused by gastric cancer. OS was defined as death regardless of reasons. Survival time was defined as time from the date of diagnosis to death or last contact or December 31, 2019.

**Construction of nomogram**

Variables were selected by three ways: 1) variables with significant level below 0.10 in univariable cox regression. 2) Best Subsets Regression (BSR)[1] which showed great benefit since all possible combination of variables were calculated and the final selected combined model owned the maximum adjusted R^2^. 3) LASSO regression and cross validation to select the combined model with optimal λ[2]. The variables chose by the above three methods were included in multivariate cox regression. Then model selection was performed by a backward stepwise process with the minimum Akaike information criterion (AIC). Finally, three models were compared by time-dependent receiver operating characteristic (ROC) curve[3]. The model with the highest area under the ROC curve (AUC) was selected and the corresponding nomogram based on the model was constructed. We analyzed the distribution of tumor size in T1 stage and chose the quartiles to be the cut-off value, which classified tumor size into four parts: ≤1.2cm; 1.2-2.1cm; 2.1-3.7cm; ≥3.7cm.

**Comparison between nomograms**

We used a series of methods to evaluate the performance between the models with tumor size included and not included. The calibration curves were used to assess the coincidence between the actual outcomes and the predicted probabilities. We used C-index and ROC curve to evaluate the discriminative ability, the net reclassification improvement (NRI) and integrated discrimination improvement (IDI) to evaluate the reclassification ability[4, 5], the decision curve analyses (DCA) [6, 7] to evaluate clinical usefulness and benefits. The NRI was category-free. Both calibration and discrimination were evaluated using bootstrapping with 1,000 resamples.

**Statistical analysis**

Univariate and multivariate cox regression models were utilized to identify variables significantly associated with CSS and OS. The discrimination ability of tumor size and other factors was assessed by the concordance index (C-index) and ROC curve.A C-index of 1 indicating perfect discrimination and a C-index of 0.5 indicating a random guess. Higher area under the ROC (AUC) was considered to show better discriminatory ability. AUC at 5 years was mainly analyzed in our study. Survival curves for CSS and OS were generated using the Kaplan–Meier method.
