# Supplementary material for: Tumor size as a significant prognostic factor in T1 gastric cancer: a Surveillance, Epidemiology, and End Results (SEER) database analysis
Source: BMC Gastroenterol. 2023 Apr 12;23:121. doi: 10.1186/s12876-023-02737-z (PMC10091636; doi:10.1186/s12876-023-02737-z)
Supplement: Supplementary file 2 — Additional file 2: Supplementary table 1. Univariate and Multivariate analysis of prognostic factors affecting OS. [file 12876_2023_2737_MOESM2_ESM.pdf]

Supplementary table 1: Univariate and Multivariate analysis of prognostic factors affecting OS

Factor	Univariate		Multivariate	
	HR(95%CI)	P value	HR(95%CI)	P value
**Age**				
**<68**	Reference	.000	Reference	.000
**≥68**	1.721	.000	1.711	.000
**Gender**				
**Male**	Reference	.003	Reference	.000
**Female**	0.934		0.894	
**Ethnicity**				
**White**	Reference	.000	Reference	.000
**Black**	1.049	.150	1.074	.070
**Other**	0.712	.000	0.798	.000
**Marital Status**				
**Single**	Reference	.000	Reference	.000
**Married**	0.852	.000	0.834	.000
**Widowed**	1.386	.000	1.086	.079
**Divorced**	0.964	.432	0.970	.551
**Site of cancer**				
**proximal**	Reference	.000	Reference	.014
**middle**	0.782	.000	0.890	.001

**distal**	0.812	.000	0.927	.051
**overlapping**	1.067	.190	0.972	.617
**Grade**				
**I**	Reference	.000	Reference	.000
**II**	1.298	.000	1.056	.376
**III/IV**	1.492	.000	1.224	.001
**Histology**				
**Adenocarcinoma**	Reference	.013	Reference	.918
**Mucinous adenocarcinoma**	1.030	.626	0.985	.828
**Signet ring cell carcinoma**	0.927	.005	0.988	.711
**T stage**				
**T1**	Reference	.000	Reference	.000
**T2**	1.265	.000	1.216	.000
**T3**	1.855	.000	1.597	.000
**T4**	2.432	.000	2.170	.000
**N stage**				
**N0**	Reference	.000	Reference	.000
**N1**	1.458	.000	1.213	.000
**N2**	1.659	.000	1.602	.000
**N3**	2.068	.000	2.179	.000

**Tumor size**	1.032	.000	1.014	.000
**Surgery**				
**Partial or subtotal or hemi-**	Reference	.000	Reference	.000
**Near-total or total**	1.089	.035	1.002	.959
**With removal of a portion of esophagus**	1.139	.000	1.171	.000
**With the resection of other organs**	1.138	.004	1.065	.202
**Surgery, NOS**	0.912	.548	1.033	.854
**Not surgery**	3.299	.000	2.421	.000
**LNH**				
**None**	Reference	.000	Reference	.000
**1-3**	0.519	.000	1.147	.112
**≥4**	0.386	.000	0.659	.000
**Unknown number**	0.408	.000	0.785	.116

HR: Hazard ratio; LNH: Lymph Node Harvest;
