# Supplementary material for: Tumor size as a significant prognostic factor in T1 gastric cancer: a Surveillance, Epidemiology, and End Results (SEER) database analysis
Source: BMC Gastroenterol. 2023 Apr 12;23:121. doi: 10.1186/s12876-023-02737-z (PMC10091636; doi:10.1186/s12876-023-02737-z)
Supplement: Supplementary file 3 — Additional file 3: Supplementary table 2. Univariate and Multivariate analysis of prognostic factors affecting CS. [file 12876_2023_2737_MOESM3_ESM.pdf]

Supplementary table 2: Univariate and Multivariate analysis of prognostic factors affecting CSS

Factor	Univariate		Multivariate	
	HR(95%CI)	P value	HR(95%CI)	P value
**Age**				
**<68**	Reference	.000	Reference	.000
**≥68**	1.427	.000	1.436	.000
**Gender**				
**Male**	Reference	.009	Reference	.100
**Female**	0.933		0.948	
**Ethnicity**				
**White**	Reference	.000	Reference	.000
**Black**	1.029	.459	1.084	.076
**Other**	0.700	.000	0.807	.000
**Marital Status**				
**Single**	Reference	.000	Reference	.000
**Married**	0.885	.000	0.874	.001
**Widowed**	1.263	.000	1.114	.048
**Divorced**	0.954	.376	0.977	.690
**Site of cancer**				
**proximal**	Reference	.000	Reference	.000
**middle**	0.706	.000	0.813	.000

**distal**	0.729	.000	0.851	.000
**overlapping**	1.021	.710	0.884	.052
**Grade**				
**I**	Reference	.000	Reference	.000
**II**	1.520	.000	1.162	.052
**III/IV**	1.940	.000	1.427	.000
**Histology**				
**Adenocarcinoma**	Reference	.792	Reference	.853
**Mucinous adenocarcinoma**	1.048	.500	0.991	.906
**Signet ring cell carcinoma**	1.006	.852	1.019	.591
**T stage**				
**T1**	Reference	.000	Reference	.000
**T2**	1.359	.000	1.243	.000
**T3**	2.415	.000	1.857	.000
**T4**	3.312	.000	2.675	.000
**N stage**				
**N0**	Reference	.000	Reference	.000
**N1**	1.728	.000	1.339	.000
**N2**	2.022	.000	1.828	.000
**N3**	2.612	.000	2.534	.000

**Tumor size**	1.034	.000	1.017	.000
**Surgery**				
**Partial or subtotal or hemi-**	0.273	.000	Reference	.000
**Near-total or total**	1.199	.000	1.039	.464
**With removal of a portion of esophagus**	1.261	.000	1.203	.000
**With the resection of other organs**	1.279	.000	1.126	.032
**Surgery, NOS**	1.003	.988	1.039	.844
**Not surgery**	3.661	.000	2.686	.000
**LNH**				
**None**	Reference	.000	Reference	.000
**1-3**	0.464	.000	1.167	.133
**≥4**	0.373	.000	0.639	.000
**Lymph nodes removed，NOS**	0.446	.000	0.867	.412

HR: Hazard ratio; LNH: Lymph Node Harvest
