# Supplementary material for: Tumor size as a significant prognostic factor in T1 gastric cancer: a Surveillance, Epidemiology, and End Results (SEER) database analysis
Source: BMC Gastroenterol. 2023 Apr 12;23:121. doi: 10.1186/s12876-023-02737-z (PMC10091636; doi:10.1186/s12876-023-02737-z)
Supplement: Supplementary file 4 — Additional file 4: Supplementary figure 2. Heatmap of C-index of clinicopathological factors in predicting CSS and OS in gastric cancer. [file 12876_2023_2737_MOESM4_ESM.pdf]

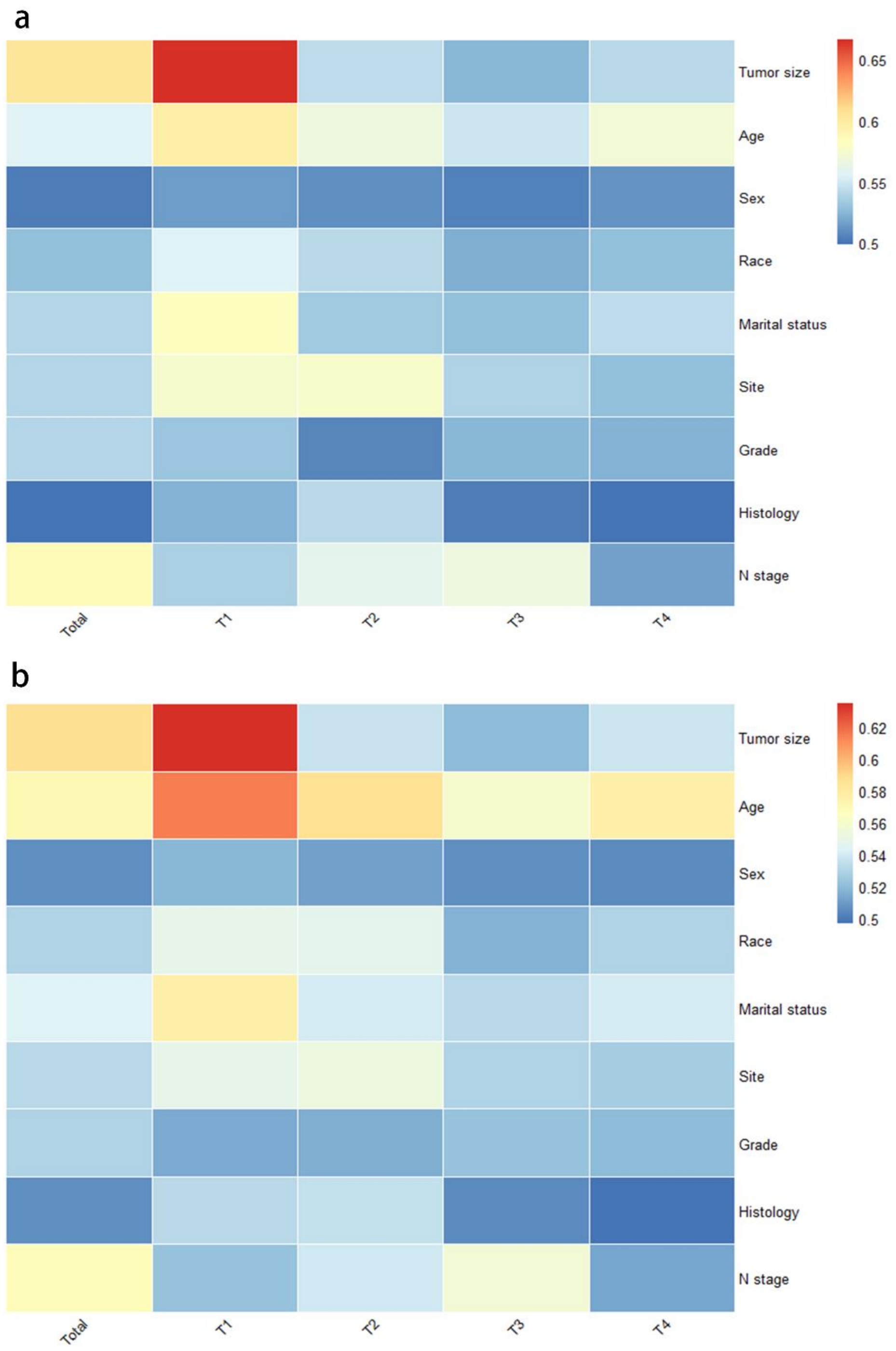

Supplementary figure 2 Heatmap of C-index of clinicopathological factors in predicting CSS and OS in gastric cancer. (a): CSS in SEER database.(b): OS in SEER database. Abbreviations: CSS, cancer-specific survival; OS, overall survival; SEER, Surveillance, Epidemiology, and End Results.
