# Supplementary material for: Tumor size as a significant prognostic factor in T1 gastric cancer: a Surveillance, Epidemiology, and End Results (SEER) database analysis
Source: BMC Gastroenterol. 2023 Apr 12;23:121. doi: 10.1186/s12876-023-02737-z (PMC10091636; doi:10.1186/s12876-023-02737-z)
Supplement: Supplementary file 5 — Additional file 5: Supplementary table 3. Discriminatory ability of clinicopathological factors in predicting OS in gastric cancer. [file 12876_2023_2737_MOESM5_ESM.pdf]

**Supplementary table 3. Discriminatory ability of clinicopathological factors in predicting OS in gastric cancer**

| | Total | | T1 | | T2 | | T3 | | T4 | |
|---|---|---|---|---|---|---|---|---|---|---|
| | C-index | AUC | C-index | AUC | C-index | AUC | C-index | AUC | C-index | AUC |
| **Tumor size** | 0.587 | 0.635 | 0.635 | 0.660 | 0.537 | 0.547 | 0.520 | 0.525 | 0.538 | 0.576 |
| **Age** | 0.571 | 0.577 | 0.615 | 0.640 | 0.586 | 0.605 | 0.560 | 0.559 | 0.576 | 0.584 |
| **Sex** | 0.506 | 0.521 | 0.519 | 0.535 | 0.511 | 0.524 | 0.506 | 0.517 | 0.505 | 0.500 |
| **Race** | 0.530 | 0.543 | 0.549 | 0.565 | 0.547 | 0.565 | 0.517 | 0.532 | 0.530 | 0.548 |
| **Marital_status** | 0.543 | 0.548 | 0.576 | 0.589 | 0.541 | 0.530 | 0.532 | 0.537 | 0.541 | 0.567 |
| **Site** | 0.532 | 0.557 | 0.549 | 0.561 | 0.553 | 0.592 | 0.530 | 0.564 | 0.526 | 0.518 |
| **Grade** | 0.530 | 0.537 | 0.514 | 0.528 | 0.515 | 0.523 | 0.522 | 0.516 | 0.520 | 0.538 |
| **Histology** | 0.506 | 0.508 | 0.532 | 0.545 | 0.535 | 0.546 | 0.505 | 0.505 | 0.497 | 0.548 |
| **N stage** | 0.568 | 0.632 | 0.523 | 0.535 | 0.539 | 0.567 | 0.557 | 0.607 | 0.513 | 0.577 |
| **Surgery** | 0.600 | 0.632 | 0.694 | 0.732 | 0.605 | 0.642 | 0.578 | 0.602 | 0.560 | 0.588 |
| **LNH** | 0.593 | 0.609 | 0.687 | 0.727 | 0.599 | 0.621 | 0.578 | 0.592 | 0.574 | 0.592 |

LNH: Lymph Node Harvest; AUC, area under the receiver operating characteristic curve （ROC）
