# Supplementary material for: Tumor size as a significant prognostic factor in T1 gastric cancer: a Surveillance, Epidemiology, and End Results (SEER) database analysis
Source: BMC Gastroenterol. 2023 Apr 12;23:121. doi: 10.1186/s12876-023-02737-z (PMC10091636; doi:10.1186/s12876-023-02737-z)
Supplement: Supplementary file 6 — Additional file 6: Supplementary table 4. C-index of clinicopathological factors in subgroup. [file 12876_2023_2737_MOESM6_ESM.pdf]

Supplementary table 4. C-index of clinicopathological factors in subgroup

	Surgery status		Lymph nodes status	
	No surgery	Performed surgery	No lymph nodes removed	lymph nodes removed
**Tumor size**	0.580	0.592	0.632	0.595
**Age**	0.543	0.569	0.551	0.577
**Sex**	0.522	0.537	0.519	0.542
**Race**	0.505	0.553	0.520	0.552
**Marital status**	0.539	0.540	0.560	0.541
**Site**	0.515	0.568	0.521	0.568
**Grade**	0.529	0.527	0.554	0.525
**Histologic**	0.506	0.520	0.511	0.525
**N stage**	0.514	0.587	0.528	0.594
**Surgery**	0.5	0.552	0.618	0.564
**LNH**	0.501	0.527	0.5	0.530

LNH: Lymph Node Harvest
