# Supplementary material for: Tumor size as a significant prognostic factor in T1 gastric cancer: a Surveillance, Epidemiology, and End Results (SEER) database analysis
Source: BMC Gastroenterol. 2023 Apr 12;23:121. doi: 10.1186/s12876-023-02737-z (PMC10091636; doi:10.1186/s12876-023-02737-z)
Supplement: Supplementary file 7 — Additional file 7: Supplementary figure 3. Survival analysis of CSS and OS stratified by tumor size. [file 12876_2023_2737_MOESM7_ESM.pdf]

Supplementary figure 3

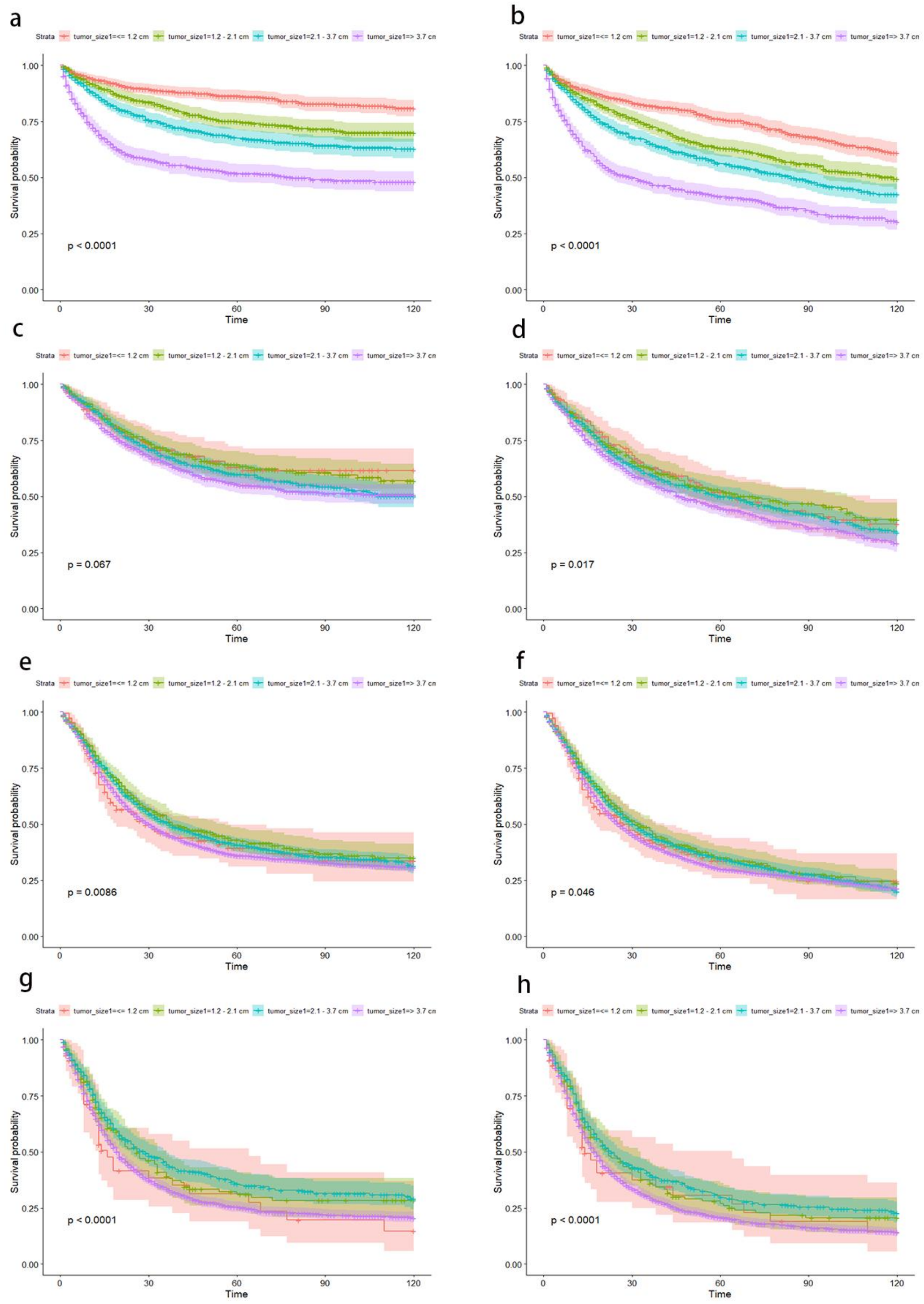

Supplementary figure 3 Survival analysis of CSS and OS stratified by tumor size. a): survival curves exhibiting CSS in T1 stage. b): survival curves exhibiting OS in T1 stage. c): survival curves exhibiting CSS in T2 stage. d): survival curves exhibiting OS in T2 stage. e): survival curves exhibiting CSS in T3 stage. f): survival curves exhibiting OS in T3 stage. g): survival curves

exhibiting CSS in T4 stage. h): survival curves exhibiting OS in T4 stage. Abbreviations: CSS, cancer-specific survival; OS, overall survival
